# Relationship between daily swallowing frequency and pneumonia in patients with severe cerebral palsy

**DOI:** 10.1186/s12887-022-03547-0

**Published:** 2022-08-13

**Authors:** Nobukazu Tanaka, Kanji Nohara, Chisato Uota, Nami Fujii, Aya Obana, Katsuji Tanaka, Takayoshi Sakai

**Affiliations:** 1 Division of Oral and Facial Disorders, Osaka University Dental Hospital, 1-8, Yamadaoka, Suita, Osaka, 565-0871 Japan; 2 Division of Functional Oral Neuroscience, Graduate School of Dentistry, Osaka University, 1-8, Yamadaoka, Suita, Osaka, 565-0871 Japan; 3 Nishinomiya-Sunago Medical and Welfare Center for Children with Severe Motor and Intellectual Disabilities, 2-9 Mukogawa, Nishinomiya, Hyogo 663-8131 Japan

**Keywords:** Cerebral palsy, Swallowing frequency, Aspiration pneumonia, Swallowing function, Dysphagia

## Abstract

**Background:**

Aspiration pneumonia is a major complication that occurs in patients with severe cerebral palsy and is associated with their survival prognosis, necessitating appropriate assessment and response. We focused on swallowing frequency as an index of daily swallowing function due to the difficulty in evaluating the risk of pneumonia. The swallowing motion protects the airway by safely directing the food, saliva, and secretions accumulated in the pharynx into the esophagus to prevent aspiration and entry into the trachea. Thus, swallowing frequency may be correlated with the incidence of pneumonia. In this study, we aimed to investigate the relationship between swallowing frequency and history of pneumonia in patients with severe cerebral palsy.

**Methods:**

Fifty-seven patients with cerebral palsy were included in this study. Swallowing frequency was measured three times for each patient on separate days, and the reproducibility was examined by calculating the intraclass correlation coefficient. Further, the relationship between swallowing frequency and history of pneumonia was investigated using multivariate logistic regression analysis.

**Results:**

While swallowing frequency differed between participants, it was constant within individuals (intraclass correlation coefficient: 0.941). Furthermore, the swallowing frequencies per hour were 12.2 ± 12.2 and 27.0 ± 20.4 in the patient groups with and without a history of pneumonia, respectively (*P* < 0.001). Swallowing frequency (odds ratio: 10.489, 95% confidence interval: 2.706–40.663, *P* = 0.001) was significantly associated with the incidence of pneumonia in the previous year.

**Conclusions:**

Swallowing frequency could be used as an index for assessing the risk of dysphagia and pneumonia in patients with severe cerebral palsy.

## Background

Cerebral palsy (CP) causes motor disturbances in the limbs and various types of dysphagia following birth. Symptoms of dysphagia appear in various places, ranging from the oral cavity to the esophagus, and the impact varies from mild to severe [1–3]. The prevalence of dysphagia is particularly high in patients with severe CP. Reportedly, 99% of these patients have dysphagia [4]. The prognosis of CP has improved in recent years owing to advances in medical technology and the quality of care. Therefore, in addition to dysphagia caused by oral motor dysfunction, abnormal neurological maturation, and anatomical abnormalities, which are characteristic of CP, age-related functional decline is now considered to exert a major impact on the prognosis and quality of life in adults with CP [5, 6]. This necessitates managing dysphagia in the daily care of patients with CP, regardless of their age.

Aspiration and aspiration pneumonia are major complications that occur in patients with CP and dysphagia. Moreover, they are directly correlated with the survival prognosis [7–9]. Thus, early and appropriate assessment and management of the aforementioned risk are extremely important. Video fluoroscopy and video endoscopy are the gold standards for detecting aspiration, particularly silent aspiration. The rate of aspiration detection using the aforementioned methods is much higher than that using other evaluation methods, even in patients with CP [10]. However, the prevalence of aspiration varies widely from 31 to 97% in such cases, despite the use of standard swallowing function tests [11–13]. In addition, the relationship between aspiration detected by examination and the incidence of pneumonia is reportedly low in older adults and patients with CP [14, 15]. The severity of aspiration in CP remains unclear; thus, the state of aspiration and the risk of pneumonia also remains unclear.

One reason for this unclarity is the difficulty in reproducing daily swallowing functions within the limited confines of an examination in patients with physical and mental disabilities, which can be attributed to the effects of complications, such as myotonia and scoliosis [16]. Moreover, the aspiration of reflux substances because of dysfunction of the upper gastrointestinal tract is a possible risk factor for pneumonia [17, 18]. Despite the efficacy of standard swallowing function tests, such as video fluoroscopy and video endoscopy in detecting aspiration during examination, it is necessary to assess the risk of pneumonia by considering the possibility of aspiration at other times and places outside the examination. The resulting data from the aforementioned studies highlight the current difficulty in evaluating pneumonia risk. This necessitates indicators that reflect daily swallowing function to assess aspiration and pneumonia risks in patients with severe CP, in addition to the results of standard testing methods.

Therefore, we focused on swallowing frequency as a new index of swallowing function. Swallowing motion prevents aspiration and facilitates the safe passage of food, saliva, and secretions accumulated in the pharynx into the esophagus without entering the trachea [19, 20]. Therefore, the frequency of swallowing—that is, the number of times the swallowing motion occurs daily—can be considered an index for evaluating the risk of regular aspiration, and by extension, pneumonia. The relationship between aspiration and swallowing frequency has been demonstrated in video endoscopy studies [21, 22]. Moreover, some studies suggested an association between swallowing frequency and pneumonia in elderly people and stroke survivors [23, 24]. However, the aforementioned reports included elderly people, post-stroke survivors, or inpatients in acute care hospitals. In addition, no studies have examined the daily swallowing frequency or the relationship between swallowing frequency and pneumonia in patients with CP.

Therefore, we aimed to measure the swallowing frequency in patients with severe CP to address the following objectives: (1) the daily swallowing frequency and (2) the association between swallowing frequency and pneumonia.

## Methods

### Study design and participants

This study included 57 patients with CP and severe motor and mental disabilities who were residents of a medical welfare center specializing in severe motor and intellectual disabilities (mean age, 44.8 ± 12.7 years; 16–65 years; male-to-female ratio, 30:27). The patient characteristics are shown in Table 1.

**Table 1:** Patient characteristics

Case	*n* = 57	
**Age (years)**		
	Mean (SD)	44.8 (12.7)
	Range	16–60
**Sex**	*n (%)*	
	Male	30 (53)
	Female	27 (47)
**Type of cerebral palsy**	*n (%)*	
	Spastic	52 (91)
	Athetoid	1 (2)
	Mixed	4 (7)
**GMFCS**	*n (%)*	
	Level III	1 (2)
	Level IV	7 (12)
	Level V	49 (86)
**FOIS**	*n (%)*	
	Level 6	2 (3)
	Level 5	5 (9)
	Level 4	16 (28)
	Level 3	10 (18)
	Level 2	3 (5)
	Level 1	21 (37)

*SD* Standard deviation

*GMFCS* Gross Motor Function Classification System

*FOIS* Functional Oral Intake Scale

We obtained consent from 68 participants. However, we eventually included 57 participants after excluding individuals who had undergone laryngeal separation or laryngectomy (*n* = 6), had received respiratory management on a ventilator (*n* = 3), and were deemed unsuitable by the attending physician, nurse, or caregiver for medical or lifestyle reasons (e.g., requiring an active treatment that created deviations from normal life at the beginning of the study) (*n* = 2).

### Measuring the swallowing frequency

Based on a report by Tanaka et al. [25], we adopted a method of measuring swallowing frequency that identified swallowing from swallowing sounds using a laryngeal microphone (SH-12ik, Nanzu, Shizuoka, Japan) attached to the neck and an integrated circuit recorder (ICD-PX240, SONY, Tokyo, Japan). This noninvasive method can collect data on laryngeal sounds by attaching a laryngeal microphone to the neck (secured in place with a band), which uses almost no restraints and places extremely few limitations on activity. Thus, we considered it suitable for measuring the daily swallowing frequency of the study participants. The conditions for measuring the swallowing frequency were those of daily life in the ward where the participants resided. We acquired the measurements for 1 h between 2:00 p.m. and 4:00 p.m. after the device was worn. The number of times of swallowing per hour was counted and used as the swallowing frequency. There were no behavioral restrictions, except that the participants were requested to refrain from eating, drinking, and bathing (showering) 30 min before the measurement. The measurement was postponed if a participant manifested acute symptoms that required medical care, such as fever or epileptic seizures on the measurement day. In addition, a supervisor regularly patrolled the ward during the measurements to check and record the participants’ behavior and status, including the status of wearing the device, and could halt the measurements at any time if deemed necessary.

We assessed each participant thrice to examine the variations in the swallowing frequency between individuals. All measurements were acquired under the aforementioned conditions, once a week for 3 consecutive weeks (i.e., a total of three measurements over 3 weeks).

### History of pneumonia

We assessed the history of pneumonia by retrospectively reviewing the medical records for pneumonia over the previous year, from the day of the first swallowing frequency measurement. Pneumonia was diagnosed by the attending physician if radiographic evidence of an infiltrate plus one or more of the following symptoms of respiratory problems were present: pyrexia (body temperature > 38 °C), cough, purulent sputum, and dyspnea or tachypnea (respiratory rate > 25/min). We considered a history of pneumonia if the patient had received a comprehensive diagnosis of pneumonia from an attending physician based on various clinical examinations, such as symptoms, blood tests, and chest imaging. We excluded pneumonia cases clearly caused by a virus or diagnosed as interstitial pneumonia.

### Compliance with ethical standards

This study was approved by Nishinomiya-Sunago Medical and Welfare Center for Children with Severe Motor and Intellectual Disabilitie ethics committee (Hyogo, Japan; approval no.: R1– E48). Before the start of the study, its content and methods were explained to the all participants’ legal guardians or caretakers, and their informed consent was obtained in writing. Participants who were capable of communicating also received an oral explanation, and only those who provided informed consent participated.

### Analytical methods

Two assessors with experience in analyzing swallowing frequency evaluated the frequency in each participant. The intra- and inter-rater reliabilities were also assessed. We used the first swallowing frequency data for each participant for these assessments. One of the assessors evaluated the intra-rater reliability by re-analyzing the data at least 1 week following the first analysis. In contrast, the inter-rater reliability was evaluated by examining the results obtained by both assessors on 29 randomly selected participants (equivalent to 50% of the participants). We avoided the introduction of bias during analysis by anonymizing the data.

We determined the variations in the swallowing frequency within individuals by calculating the intraclass correlation coefficients (ICCs) for the three swallowing frequency measurements for each participant as part of the intra-rater correlation assessment.

### Statistical analyses

We examined the relationship between swallowing frequency and pneumonia by comparing the swallowing frequency of participants with and without a history of pneumonia. We used the mean of three measurements for each participant. The groups were compared using the Mann–Whitney U test. To determine the influence of each factor on the incidence of pneumonia, we conducted a logistic regression analysis using age, sex, the presence of oral feeding, and swallowing frequency as the explanatory variables. In contrast, the presence of pneumonia history was used as the objective variable. The presence of oral feeding was based on the Functional Oral Intake Scale [26]. No patients corresponded to level 7 (total oral diet with no restrictions). Patients who corresponded to levels 6 (total oral diet, but with specific food limitations) to 3 (comprising patients who regularly ate at least one meal per day) were classified as “oral intake,” whereas patients who corresponded to levels 1 and 2 (level 2 comprised patients who ate orally with limited frequency) were classified as “no oral feeding.” We classified the frequency of swallowing into two groups based on the median (15.3 times) of all participants. Multivariate logistic regression was performed with the forced entry of variables that were significant in the univariate logistic regression analysis. All tests were two-tailed, and the significance level was set at *P* < 0.05. The data were analyzed with no imputation of the missing values or the removal of outliers. We used SPSS version 22.0 for Windows (IBM Corp., Armonk, NY, USA) for the analysis. The analysis method was selected, and the analyses were conducted by a professional statistical analysis company (Statista, Kyoto, Japan).

## Results

### Swallowing frequency

The ICCs for the intra- and inter-rater reliability were 0.981 for ICC (1,1) and 0.936 for ICC (2,1), respectively, demonstrating a high degree of correlation within and between the assessors. The mean swallowing frequency per hour for all participants was 21.3 ± 22.6 times (range: 1–111 times), based on a total of 171 measurements obtained from 57 participants. In contrast, the overall ICC (1,1) was 0.941. The ICC (1,1) for the presence and absence of pneumonia history was 0.947 and 0.927, respectively, displaying a high degree of correlation within the groups (Table 2).

**Table 2:** Intraclass correlation coefficients (1,1) and standard error

	Intraclass correlation coefficient			Variation	
	N	ICC (1,1)	95% confidence interval	Standard error	MDC95
Overall	57	0.941	0.910, 0.963	5.6	15.5
History of pneumonia	22	0.947	0.947, 0.976	5.4	15.0
No history of pneumonia	35	0.927	0.927, 0.959	5.7	15.7

*ICC* Intraclass correlation coefficients

*MDC95* Minimal detectable change 95

### Relationship between swallowing frequency and history of pneumonia in the previous year

**Fig. 1:**
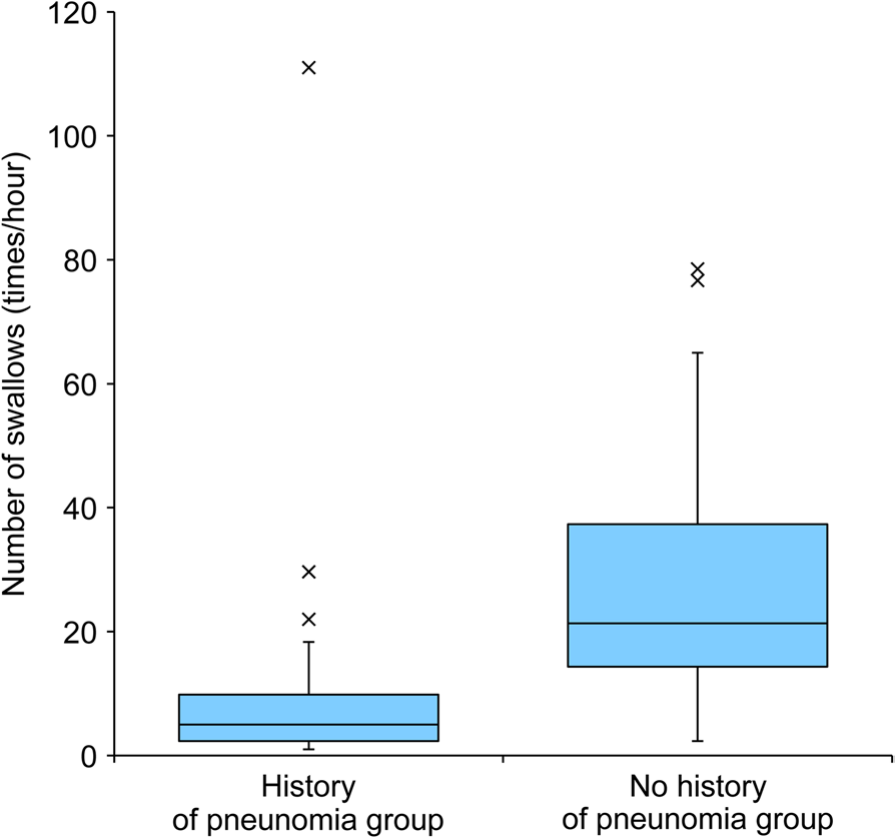
Comparison by presence or absence of pneumonia history. Left label: History of pneumonia group. Right label: No history of pneumonia group. Cross marks: Outliers

The frequency of swallowing per hour in the group that experienced pneumonia in the past year and in the group that did not was 12.2 ± 12.2 and 27.0 ± 20.4 times, respectively (Fig. 1, Table 3). Furthermore, the presence or absence of oral feeding and the number of swallows were significant in the univariate analysis. In the multivariate logistic regression analysis, the number of swallows (odds ratio, 10.489; 95% confidence interval, 2.706–40.663; *P* = 0.001) was significantly associated with the incidence of pneumonia in the past year (Table 4).

**Table 3:** Comparison of swallowing frequency (mean of three times) with and without pneumonia

	N	No pneumonia	n	Pneumonia in past year	*P*-value
Number of swallows	35	21.3 [13.3, 38.3]	22	5.0 [2.3, 10.8]	***P*** **< 0.001**

Data expressed as median [interquartile range]

*P*-value, Mann–Whitney U test

**Table 4:** Logistic regression analysis of the history of pneumonia

		Univariate			Multivariate		
	n	OR	95% CI	*P*-value	OR	95% CI	*P*-value
Age (per 1 year)	57	1.016	0.972, 1.061	0.487	-		
Sex F (vs. M)	57	1.187	0.408, 3.455	0.752	-		
Tube (vs. oral)	57	4.375	1.404, 13.636	**0.011**	2.778	0.749, 10.307	0.127
No. of swallows < median 15.3 (vs. ≥median)	57	13.000	3.465, 48.774	***P*** **< 0.001**	10.489	2.706, 40.663	**0.001**

*OR* Odds ratio, *95% CI* 95% confidence interval

Multivariate model coefficient chi-squared test: *P*=0.009

## Discussion

### Swallowing frequency

This novel study investigated the relationship between daily swallowing frequency and pneumonia in patients with severe CP. Swallowing frequencies in daily life were distributed over a wide range from 1 to 111 times per hour. While the previously reported minimum and maximum swallowing frequencies differed from our results, previous studies on elderly and healthy participants also observed individual differences in the daily swallowing frequency (203–1008 times per day Lear et al. [27] 2–76 times per hour Tanaka et al. [28]). This is similar to the individual differences we observed in patients with CP.

Swallowing frequency may be affected by various factors, specifically wakefulness, sleep depth, saliva volume, and drooling [29–35]. Our objective was to measure the daily swallowing frequency during non-meal times. While the participants included those with poor wakefulness or drowsiness, we considered these to be among the factors that contributed to their “regular state” and may affect the swallowing frequency. Thus, we did not use the aforementioned factors as the exclusion criteria. However, we acquired three measurements to check for variation in the swallowing frequency within individuals in states that included these factors. The ICC results of the intra-rater correlation assessments displayed a high degree of correlation.

Therefore, the swallowing frequency of each participant varied little on a daily basis, that is, it remained constant. The lack of extreme variability in the daily swallowing frequency within each participant was useful as an indicator for functional or risk assessment.

The attending physicians determined the presence of pneumonia based on the medical records and the results of various clinical and imaging examinations. It is difficult to confirm whether all cases were of the aspiration type based on its definition [36], which is a limitation of this study. Despite the variations in severity, all patients had CP with reduced swallowing functions. Hence, it is probable that aspiration was involved in causing pneumonia.

The swallowing frequency in patients with pneumonia in the previous year was significantly lower than that in patients without a history. Moreover, the multivariate analysis established an association between reduced swallowing frequency and pneumonia. Reduced swallowing frequency supposedly affects swallowing functions through the disuse of swallowing-related organs or secondary sarcopenia of the related muscles. Reduced swallowing frequency among the study participants indicated that their swallowing-related organs were moving less frequently, and a chronic state was likely to cause disuse or sarcopenia. A previous study primarily involving the elderly reported that those with aspiration (aspirators) exhibited greater loss of lingual and suprahyoid muscle strength and mass than the regular age-related decline [37–39]. The geniohyoid muscle is involved in elevating the larynx and is particularly prone to disuse and sarcopenia. Moreover, limitations on oral feeding promote (cause) atrophy of this muscle [40]. Functional decline in the muscles involved in elevating the larynx could be a direct cause of aspiration during the swallowing motion, which eventually hinders the appropriate timing and the amount of opening to the esophagus. Despite the patients having severe CP, we anticipated that they could experience a similar phenomenon as the elderly participants. Therefore, the decrease in the daily swallowing frequency could promote a functional decline in the swallowing-related muscles over the long term. This, in turn, could eventually result in aspiration-mediated pneumonia. Further studies are required to determine whether swallowing frequency causes the disuse of swallowing-related muscles.

Reduced swallowing frequency can increase the risk of aspiration. In CP cases, pneumonia is caused by the aspiration of food [17, 41], saliva, pharyngeal secretions [15, 42], and refluxed contents of the stomach and esophagus, including gastric acid [17, 42]. Swallowing movement is an airway defense mechanism that clears particles that have flowed into or accumulated in the pharynx by directing them to the esophagus and preventing them from entering the pharynx. This defense mechanism is effective against the anterograde aspiration of ingested food and secreted saliva and the retrograde aspiration of reflux substances [19]. Therefore, swallowing frequency reflects the state of the defense mechanism against aspiration. Previous studies [21, 22] on the elderly have reported that cases with reduced swallowing frequency have increased retention of secretions in the pharynx and greater food aspiration. In addition, reduced swallowing frequency is likely to increase the risk of aspiration. In the present study, the above-mentioned mechanism contributed to the decrease in the swallowing frequency and the history of pneumonia. Our results suggested that reduced swallowing frequency increases the risk of aspiration, even in patients with CP. In addition, the degree of swallowing frequency may be a useful index for the effective functioning of the airway defense mechanism against aspiration. However, the patients in this study were a group of individuals with a decline in swallowing function, and it was not possible to evaluate the presence or absence of aspiration by instrumental evaluation of swallowing in a few patients. Therefore, further consideration is needed to clarify these findings.

Considering the difficulty in evaluating the daily swallowing status with standard swallowing function tests, such as video fluoroscopy and video endoscopy, the frequency of swallowing may be a useful index for evaluating decreased swallowing function and the risk of pneumonia in children with disabilities who are prone to aspiration of reflux substances. Appropriate assessments are imperative for the management of food-related issues. The safe and continuous maintenance of oral feeding is essential for increasing survival prognosis and improving the quality of life of patients with CP and reduced swallowing function.

While we examined the association between swallowing frequency and history of pneumonia, further investigation is required to determine whether swallowing frequency can predict the onset of pneumonia. We intend to conduct a prospective study to determine the appropriate cutoff values for swallowing frequency and consider the effects of aspiration and reflux using instrumental evaluations of these symptoms.

## Conclusions

This study showed that (1) daily swallowing frequency differs among patients with CP but is constant in each of them, and 2) a decrease in swallowing frequency is associated with pneumonia. These results suggest that the daily swallowing frequency may be useful as an indicator of the risk of pneumonia.

## Data Availability

The datasets used and analyzed during the current study are available from the corresponding author on reasonable request.

## Abbreviations

CP: Cerebral palsy
ICC: Intraclass correlation coefficient
